# Amoxicillin-associated hemorrhagic colitis: A case report and literature review

**DOI:** 10.1097/MD.0000000000040800

**Published:** 2024-12-06

**Authors:** Yu-Ling Xiong, Chao Peng, Ying-Jiang Deng, Wei Li, Yin Huang, Yue Tian

**Affiliations:** a Department of Gastroenterology, Fengdu People’s Hospital, Chongqing, China; b Department of Traditional Chinese Medicine and Rheumatology and Immunology, Fengdu People’s Hospital, Chongqing, China; c Department of Pathology, Fengdu People’s Hospital, Chongqing, China.

**Keywords:** abdominal pain, amoxicillin, antibiotic-associated, bloody stools, hemorrhagic colitis

## Abstract

**Rationale:**

Antibiotic-associated hemorrhagic colitis (AAHC) is a special type of antibiotic-associated colitis. Due to the increased use of antibiotics, especially amoxicillin, which is commonly used in clinical practice, the incidence of antibiotic-associated hemorrhagic colitis has also increased. However, doctors have insufficient understanding of this disease, and patients may be missed or misdiagnosed.

**Patient concerns:**

A 71-year-old female patient was admitted to our hospital with abdominal pain, diarrhea, and bloody stools. There was a history of oral amoxicillin before the onset of the disease.

**Diagnosis:**

The final diagnosis of this patient was amoxicillin-associated hemorrhagic colitis.

**Interventions:**

The patient stopped using antibiotics and was given “Bifidobacterium quadruplex live bacterial tablets and L-glutamine sodium gualenate granules” orally.

**Outcomes:**

After oral administration, the patient’s symptom rapidly were completely alleviate. Follow-up colonoscopy revealed normal mucosal images.

**Lesson:**

Through this case report, doctors should increase their understanding of the disease, especially for patients with sudden abdominal pain accompanied by bloody stools as the main complaint, it is significant to attend to inquiries about the history of antibiotic use, such as amoxicillin, and pay attention to the discovery of acid-producing *Klebsiella* in the fecal microbiota.

## 1. Introduction

Antibiotic-associated hemorrhagic colitis (AAHC) is an antibiotic-related disease that occurs after the use of antibiotics, leading to dysbiosis of the gut microbiota, secondary bacterial infections, excessive growth of acid-producing *Klebsiella pneumoniae*, and intestinal inflammation and bleeding. Due to the increased use of antibiotics, especially amoxicillin, which is commonly used in clinical practice, the incidence of AAHC has also increased. However, doctors have insufficient understanding of this disease, and patients with sudden abdominal pain and rectal bleeding may be missed or misdiagnosed. This article reports that the Fengdu People’s Hospital, Chongqing, with abdominal pain, diarrhea, and bloody stools as the first symptoms. There was a history of oral amoxicillin taken 3 days before the onset of the disease. A case of AAHC was diagnosed by combining colonoscopy, biopsy, and abdominal CT. After discontinuation of amoxicillin, auxiliary support treatment was given to rapidly alleviate symptoms.

## 2. Case presentation

A 71-year-old female patient was admitted to the Gastroenterology Department of Fengdu People’s Hospital in Chongqing on January 31, 2024, due to abdominal pain, diarrhea for 1 day, and blood in the stool for 1 hour. The patient had a sore throat and runny nose 3 days prior. They went to the pharmacy to purchase amoxicillin capsules (Southwest Pharmaceutical, 0.5 g/time, 3 times/day) and Lianhua Qingwen granules for oral use. Afterwards, their cold symptoms improved. One day prior, there was paroxysmal colic around the navel, accompanied by diarrhea, with yellow paste-like or watery stools. The frequency was frequent, and the specific details are unknown. Abdominal pain did not improve after defecation. Self-purchased “Montmorillonite Powder” and “Gastrointestinal Ning Tablets” did not improve symptoms. One hour prior, there was blood in the stool, which was a bright red bloody stool with mucus. When the water was flushed, it was visible as red and had a washed meat-like appearance 3 to 5 times. The total amount of disease, accompanied by urgency, heaviness, anal prolapse, and no fever, vomiting, purpura, joint pain, etc, is unknown. There is no special history in the past. The physical examination of the patient at admission revealed the following: soft abdomen; no abdominal wall varicose veins; gastrointestinal type or peristaltic waves; slight tenderness around the navel; no rebound pain or muscle tension; liver, spleen, or rib not palpable; negative mobile dullness; and bowel sounds 3 times/min. Anal digital examination revealed blood stains on the retracted fingertips.

**Figure 1.**
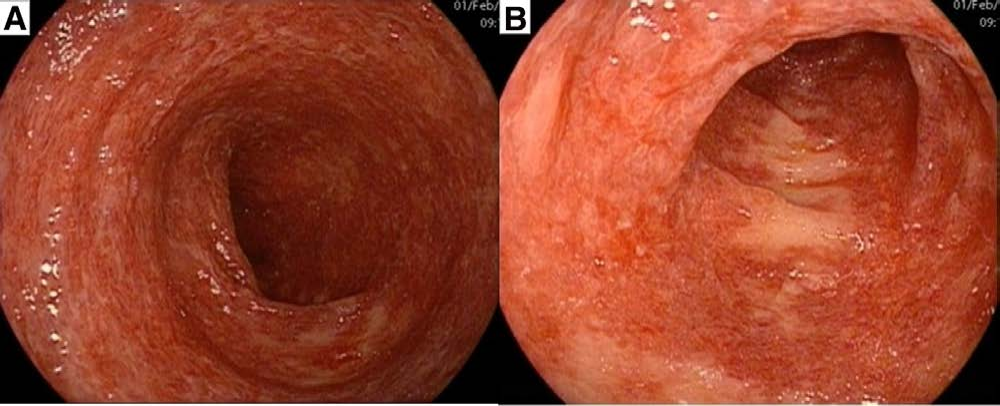
Colonoscopy examination: Widespread congestion and edema of the transverse colon mucosa, with the lesion being more severe on the oral side and affecting the entire circumference. (A) The transverse colon is 40 to 50 cm away from the anus; (B) the transverse colon is 50 to 60 cm away from the anus.

**Figure 2.**
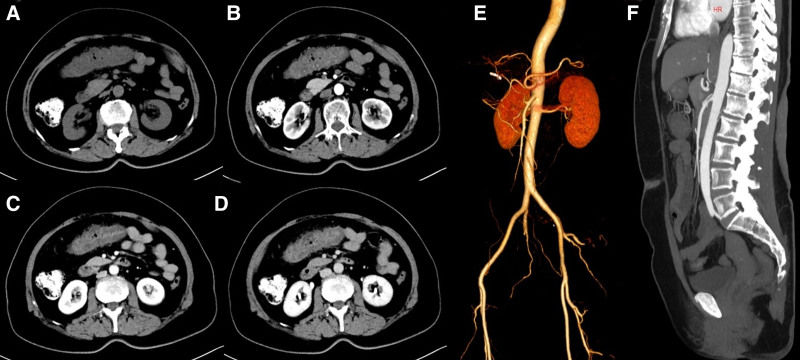
Full abdominal CT plain scan, also known as dynamic contrast-enhanced scan (coronary reconstruction image). (A) Plain scan showing edema and thickening of the transverse colon wall, with blurred fat spaces around it; (B) enhancement, arterial phase; (C) enhancement, portal phase; (D) enhancement, delay period; (B, C and D) dynamic enhanced scanning, no abnormal enhancement observed in the third phase tube wall; (E, F) three-dimensional reconstruction, no intestinal vascular stenosis or thrombosis observed.

**Figure 3.**
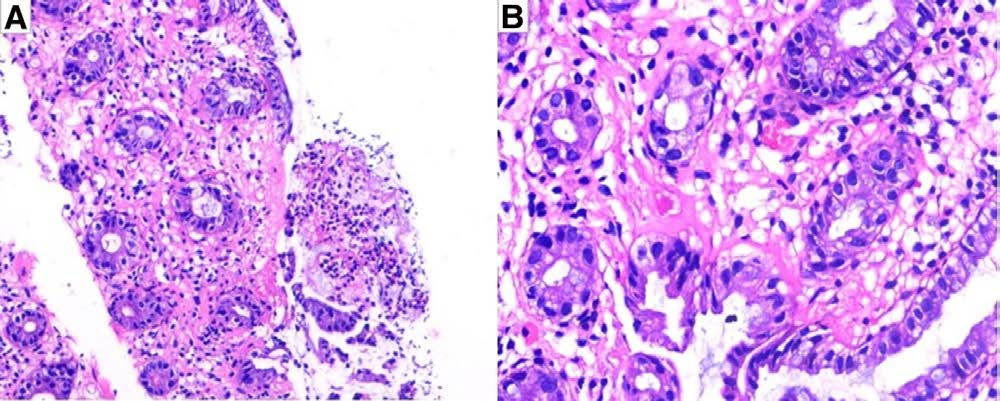
Pathological examination results of small mucosal tissue in the transverse colon (× 400). (A) Red blood cells can be seen in the lamina propria of the mucosa; (B) inflammatory cell infiltration in the mucosal lamina propria.

Written informed consent from both the patient and her kin. All relevant examinations were performed after admission. Blood parameters were as follows: white blood cell count, 12.0 × 10^9^/L; neutrophil percentage, 77.4%; red blood cell count, 4.04 × 10^12^/L; hemoglobin concentration, 127 g/L; platelet count, 145 × 10^9^/L; and hypersensitive C-reactive protein level, 7.04 mg/L. Stool routine: Red and loose stools, with 260 to 276 red blood cells/HP and 3 to 11 white blood cells/HP, no pus cells or phagocytes, and no parasites or insect eggs. Fecal culture: No pathogenic bacteria, such as *Salmonella*, *Shigella*, or pathogenic *Escherichia coli*, were detected, and no acid-producing *Klebsiella* or *Clostridium difficile* were found. On the day of admission, colonoscopy examination revealed extensive congestion and edema of the intestinal mucosa at a distance of 40 to 60 cm from the anus (transverse colon), with visible mucus attachment on the surface. The lesion was most severe at a distance of 50 to 60 cm from the anus, affecting the entire circumference. The endoscopic diagnosis was colitis: medication? Ischemia? (Fig. 1). Further emergency full abdominal enhanced CT revealed edema and thickening of the intestinal wall of the transverse colon, blurred surrounding fat spaces, and no abnormal enhancement on the enhanced scan, suggesting inflammatory lesions (Fig. 2). On February 1st, the pathological biopsy revealed infiltration of red blood cells and inflammatory cells in the mucosal lamina propria of the transverse colon (Fig. 3).

**Figure 4.**
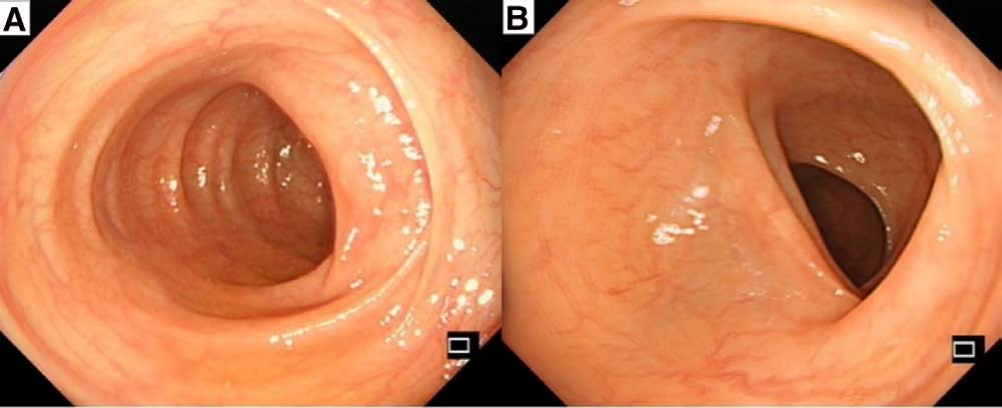
The mucosa from the rectum to the ileocecal region is smooth, and the vascular texture is clear.

The patient suffered from acute abdominal pain and bloody stool. He had no basic disease, such as hypertension, diabetes, coronary heart disease, or atrial fibrillation, and no intestinal vascular stenosis or thrombosis was found on abdominal CT. Ischemic bowel disease was temporarily considered. Considering that the patient had a history of taking amoxicillin orally before the onset of the disease and that colonoscopy revealed lesions involving the transverse colon, causing peripheral mucosal redness and edema, amoxicillin-related hemorrhagic colitis was considered. The patient stopped using antibiotics and was given “Bifidobacterium quadruplex live bacterial tablets (3 times a day, 3 tablets each time) and L-glutamine sodium gualenate granules (3 times a day, 1 tablet each time)” orally. After oral administration, the patient’s abdominal pain and rectal bleeding gradually improved. On the 5th day of hospitalization, the symptoms were completely relieved, and the patient was discharged. Follow-up for 1 month after discharge revealed no further abdominal pain, diarrhea, or bloody stools. Follow-up colonoscopy revealed normal mucosal images (Fig. 4).

## 3. Discussion

AAHC is a special type of antibiotic-associated colitis that is a rare but important complication of antibiotic treatment. It was first reported in 1978.^[1]^ The etiology of this disease is currently unclear, and several mechanisms have been proposed, including allergic reactions,^[2]^ mucosal ischemia,^[1]^ and microbial substitution induced by antibiotic-induced overgrowth of *K. pneumoniae*.^[3]^

Currently, it is widely believed that AAHC may be caused by bacterial dysbiosis and secondary infections in patients after antibiotic treatment, with the most important being *Klebsiella* and *C. difficile*. In the past decade, due to the increased use of antibiotics, the incidence of AAHC has increased. Although most patients develop secondary infections with *C. difficile*,^[4]^ animal experiments have shown^[5]^ that rats inoculated with *K. pneumoniae* without exposure to antibiotics cannot colonize and cause disease in the intestine. Rats infected with *K. pneumoniae* can develop AAHC in the right colon, while uninfected rats cannot. This indicates that *K. pneumoniae* is a pathogen of AAHC. Therefore, it is generally believed that *C. difficile* infection is not the true cause of AAHC, and *K. pneumoniae* is the main pathogenic factor.^[6,7]^

There are reports that low concentrations of amoxicillin or amoxicillin clavulanic acid can inhibit most bacteria in the gut microbiota, but concentrations that are too low cannot inhibit acid-producing *K. pneumoniae*. Due to the natural production of beta lactamases by acid-producing *K. pneumoniae*, this bacterium can resist all broad-spectrum antibiotics. Amoxicillin is a semisynthetic penicillin broad-spectrum β-lactam antibiotic, while clavulanic acid has a penicillin-like β-lactam structure that can block the active site of β-lactamase. In this case, the use of broad-spectrum antibiotics not only fails to kill *K. pneumoniae* but also may cause damage to the intestines, such as antibiotics (such as amoxicillin or amoxicillin clavulanic acid). After killing other bacteria, the activity of *K. pneumoniae* increases, resulting in high concentrations of cytotoxic toxins (such as enterotoxins and tilymcin), causing colitis, erosion, mucosal damage, etc, which leads to the occurrence of AAHC during treatment.^[8]^

This disease often presents with acute onset and usually manifests as abdominal cramps, bloody diarrhea, or bloody stools. The symptoms are severe, and systemic manifestations are rare or absent. It is easily misdiagnosed as lower gastrointestinal bleeding or chronic intestinal disease. These symptoms usually appear 2 to 3 days (up to 5 days) after the use of amoxicillin, but patients recover quickly 3 to 5 days after the discontinuation of amoxicillin.^[9]^

A typical AAHC colonoscopy examination revealed diffuse bleeding and edema of the colon mucosa, which may cause erosion and ulcer formation, but without a pseudomembrane. The bright red color of the mucosa caused by mucosal bleeding is a characteristic of this disease. The lesion is mainly located in the transverse colon, but some scholars believe that in contrast to colitis caused by *C. difficile*, colitis caused by *K. pneumoniae* is usually segmental and mainly located in the right colon (β-lactamase inhibitors have low concentrations in the right colon, and oxytocin can overgrow, leading to colitis).^[10,11]^ Pathological examination revealed that only red blood cells infiltrated the colon mucosa, and red blood cells infiltrated the intestinal cavity. AAHC has self-limiting effects, and the primary principle is to discontinue suspicious antibiotics and provide auxiliary supportive treatments, such as regulating the gut microbiota and repairing the intestinal mucosa.

Nonsteroidal anti-inflammatory drugs, anticoagulants, 5-fluorouracil, azathioprine, and other drugs are also causes of hemorrhagic colitis in clinical practice.^[11]^ Before diagnosing AAHC, it is necessary to exclude patients with a history of using these drugs. In clinical practice, it is also necessary to differentiate infectious diarrhea, pseudomembranous enteritis or *C. difficile*-related diarrhea, ischemic colitis, inflammatory bowel disease, hemorrhagic colitis caused by allergic purpura, and other diseases that cause abdominal pain and/or bloody stools. The use of relevant auxiliary examinations, such as colonoscopy, abdominal CT, fecal culture, and colon tissue, can improve the ability to distinguish and diagnose this disease, thereby avoiding misdiagnosis.

In summary, AAHC is an antibiotic-related disease that occurs after taking amoxicillin or amoxicillin clavulanic acid, leading to dysbiosis of the gut microbiota, reduced richness and diversity, excessive growth of acid-producing *K. pneumoniae*, and subsequent intestinal inflammation and bleeding.^[12]^ After discontinuation of medication, the gut microbiota can be restored to its premedication state.^[13]^ *K. acidogenes* is a gram-negative facultative *Escherichia coli* that is detected in up to 38% of normal adult feces. It is resistant to various antibiotics and has a prevalence rate of approximately 2% to 24%. It is currently becoming an important opportunistic pathogen^[14,15]^ that can cause various infections, especially AAHC. Toxins produced by certain strains of acid-producing *K. pneumoniae* (*K. oxytoca* strain) isolated from the feces of AAHC patients can cause epithelial cell death.

In China, amoxicillin is not only used for respiratory infections but also as a first-line medication for Hp treatment and is widely used in clinical practice. Some patients may have abdominal pain, diarrhea, or even bloody stool after using amoxicillin. However, due to doctors’ lack of understanding of AAHC, the disease is often misdiagnosed or missed, and its incidence rate is often higher than that reported in the literature. Therefore, for patients with sudden abdominal pain accompanied by bloody stools as the main complaint, it is important to pay attention to inquiries about the history of antibiotic use, such as amoxicillin. At the same time, doctors should increase their understanding of the disease, treat patients taking antibiotics with caution, and pay attention to the discovery of acid-producing *Klebsiella* in the fecal microbiota.

## Acknowledgments

We are very grateful to the teachers of Fengdu People’s Hospital in Chongqing for their support and Mrs. Zhang’s cooperation.

## Author contributions

**Data curation:** Yu-Ling Xiong, Chao Peng.

**Project administration:** Yu-Ling Xiong, Yue Tian.

**Resources:** Ying-Jiang Deng, Wei Li, Yin Huang.

**Writing – original draft:** Yu-Ling Xiong, Chao Peng.

**Writing – review & editing:** Yu-Ling Xiong, Yue Tian.
